# Hepatitis B Vaccine Non-Responders Show Higher Frequencies of CD24^high^CD38^high^ Regulatory B Cells and Lower Levels of IL-10 Expression Compared to Responders

**DOI:** 10.3389/fimmu.2021.713351

**Published:** 2021-09-10

**Authors:** Nina Körber, Laureen Pohl, Birgit Weinberger, Beatrix Grubeck-Loebenstein, Andrea Wawer, Percy A. Knolle, Hedwig Roggendorf, Ulrike Protzer, Tanja Bauer

**Affiliations:** ^1^Institute of Virology, Helmholtz Zentrum München, Munich, Germany; ^2^German Center for Infection Research (DZIF), Munich, Germany; ^3^Institute for Biomedical Aging Research, Universität Innsbruck, Innsbruck, Austria; ^4^Occupational Health Unit, School of Medicine, Technical University of Munich (TUM), Munich, Germany; ^5^Institute of Molecular Immunology and Experimental Oncology, School of Medicine, Technical University of Munich (TUM), Munich, Germany; ^6^Institute of Virology, School of Medicine, Technical University of Munich (TUM), Munich, Germany

**Keywords:** regulatory B cells, IL-10 expression, vaccine non-responsiveness, hepatitis B vaccination, B10^+^ cells

## Abstract

**Background:**

The cellular mechanisms involved in the lack of protective antibody response after hepatitis B vaccination are still rather unclear. Regulatory B cells (Breg) known as modulators of B-and T-cell responses may contribute to poor vaccine responsiveness. The current study aimed to investigate the role of regulatory B cells (Breg) in hepatitis B vaccine non-responsiveness after immunization with second- or third-generation hepatitis B vaccines.

**Method:**

We performed comparative phenotypic and frequency analysis of Breg subsets (CD24^+^CD27^+^ and CD24^high^CD38^high^ Breg) in second-generation hepatitis B vaccine non-responders (2^nd^ HBvac NR, n = 11) and responders (2^nd^ HBvac R, n = 8) before (d0), on day 7 (d7), and 28 (d28) after booster vaccination. Cryopreserved peripheral blood mononuclear cells were stimulated *ex vivo* with a combination of CpG, PMA, and Ionomycin (CpG+P/I) and analyzed for numbers and IL-10 expression levels of Breg by flow cytometry-based analyses.

**Results:**

Flow cytometry-based analyses revealed elevated frequencies of CD24^+^CD27^+^ Breg at all time points and significantly higher frequencies of CD24^high^CD38^high^ Breg on d0 (*p* = 0.004) and 28 (*p* = 0.012) in 2^nd^ HBvac NR compared to 2^nd^ HBvac R. In parallel, we observed significantly lower levels of CpG+P/I-induced IL-10 expression levels of CD24^+^CD27^+^ and CD24^high^CD38^high^ Breg (d0: *p* < 0.0001; d7: *p* = 0.0004; d28: *p* = 0.0003 and d0: *p* = 0.016; d7: *p* = 0.016, respectively) in 2^nd^ HBvac NR compared to 2^nd^ HBvac R before and after booster immunization.

Frequencies of CD24^+^CD27^+^ and CD24^high^CD38^high^ Breg significantly decreased after third-generation hepatitis B booster vaccination (d7: *p* = 0.014; d28: *p* = 0.032 and d7: *p* = 0.045, respectively), whereas IL-10 expression levels of both Breg subsets remained stable.

**Conclusion:**

Here we report significantly higher frequencies of CD24^high^CD38^high^ Breg in parallel with significantly lower IL-10 expression levels of CD24^+^CD27^+^ and CD24^high^CD38^high^ Breg in 2^nd^ HBvac NR compared to 2^nd^ HBvac R. Anti-HBs seroconversion accompanied by a decrease of Breg numbers after booster immunization with a third-generation hepatitis B vaccine could indicate a positive effect of third-generation hepatitis B vaccines on Breg-mediated immunomodulation in hepatitis B vaccine non-responders.

## 1 Introduction

According to the WHO 260 million people are chronically infected with HBV and 887,000 people are dying each year due to hepatitis B virus (HBV) infection (1). Second-generation hepatitis B vaccines composed of the small HBV envelope protein (hepatitis B surface antigen; HBsAg) are currently used for universal vaccination and reduce the overall incidence of both hepatitis B and the associated long-term consequences such as chronic hepatitis B and liver cirrhosis (2, 3).

Vaccination with recombinant HBsAg triggers the production of anti-HBs with an anti-HBs titer of > 10 IU/L being a very reliable surrogate marker for vaccine-induced protective immunity (4). Approximately 5-10% of vaccinees are defined as “non-responders”, i.e. they do not develop a protective anti-HBs titer after completing a full primary series of the hepatitis B vaccine (5–8). Third-generation hepatitis B vaccines containing additional HBV envelope proteins (pre-S1 and pre-S2) are known to be more immunogenic and superior in inducing protective antibody titers also in non-responders to the conventional vaccines (9). Despite the success of universal immunization programs leading to high regional vaccination coverage rates in most Western countries, non-responsiveness to hepatitis B vaccination is a major problem, especially for health care workers for whom successful hepatitis B vaccination is mandatory (10, 11). Genetically determined resistance, advanced age, gender, obesity, smoking, chronic/systemic disease, and immunosuppressive therapies are known factors contributing to non-response to immunization (6, 12–18). Several aspects like failure of antigen presentation or costimulatory signals, defects in the generation of HBsAg-specific CD4 T helper (Th) cells and insufficient production of Th1 and Th2 cytokines upon hepatitis B vaccination have been discussed, but the exact underlying immunological and molecular mechanisms contributing to hepatitis B vaccine non-responsiveness remain largely unclear (10, 19–24). Further studies investigating immune cells and immunological mechanisms involved in non-responsiveness to vaccines are urgently needed and will help to improve immunogenicity of existing or the development of new vaccines to overcome non-responsiveness to hepatitis B vaccines.

In the last years, studies revealed that regulatory B cells (Breg) have an immunosuppressive capacity and help to maintain immunological homeostasis (25, 26). Breg suppress immunopathology by skewing T-cell differentiation, induction and maintenance of regulatory T cells, as well as suppression of pro-inflammatory cells, mainly mediated through regulatory cytokines IL-10, TGF-ß, and IL-35 (25–29). There is an ongoing debate on the phenotypic characterization of different Breg subsets, specific Breg markers, and the question whether all B cells can acquire suppressive function in response to environmental triggers, or whether Breg represent a distinct lineage (28, 30–32). CD24^+/high^CD27^+^ and CD24^high^CD38^high^ Breg have been described as distinct Breg subpopulations and investigated in different disease entities like autoimmune diseases (e.g. multiple sclerosis, rheumatoid arthritis, systemic lupus erythematosus, etc.) and cancer (25, 30, 33–35). For both, CD24^+/high^CD27^+^ and CD24^high^CD38^high^ Breg it is known that they can suppress effector CD4 T cells and dendritic cells, and CD24^high^CD38^high^ Breg can additionally induce regulatory T cells (Treg) and suppress virus-specific CD8 T cells (30, 33, 36).

Rosser et al. (37) postulated three potential mechanism for Breg-mediated suppression of antibody responses, which could also play a role in the case of hepatitis B vaccine non-responsiveness: (i) Breg may alter the cytokine microenvironment in which plasma cell maturation takes place; (ii) Breg could suppress CD4 Th cells, which leads to a diminished maturation of B cells into antibody producing plasma cells; (iii) Breg could induce Treg which may contribute to an indirect suppression of antibody production (25, 37).

In the last years, some studies investigated the role of Breg in hepatitis B vaccine non-responsiveness, but findings were contradictory and in-depth analyses remain elusive (12, 38, 39). Bolther et al. concluded that levels of regulatory B cells do not predict serological responses to hepatitis B vaccination (38). In contrast, Garner-Spitzer et al. reported clearly elevated frequencies of CD24^high^CD38^high^ Breg in hepatitis B vaccine non-responders which might contribute to the increased baseline levels of IL-10 in these individuals and also lead to an induction of regulatory T cells (39).

The current study aimed to investigate different Breg subpopulations in hepatitis B vaccine responders and non-responders before and after booster vaccination with a second- *versus* a third-generation hepatitis B vaccine.

## 2 Materials and Methods

### 2.1 Study Cohorts

Two groups of hepatitis B vaccinated individuals were enrolled in this study. The first group comprised eleven non-responders to second-generation hepatitis B vaccine (2^nd^ HBvac NR) (9 women, 2 men; average age of 25.7 years) (**Table 1**). All 2^nd^ HBvac NR subjects received a full primary series of hepatitis B vaccine and were repeatedly vaccinated with Engerix^®^ or Twinrix^®^ in the past, without developing protective anti-HBs levels (> 10 IU/L). The second group consists of eight responders to second-generation hepatitis B vaccine (2^nd^ HBvac R) (6 women, 2 men; average age: 30.0 years), who received a full series of Twinrix^®^ vaccination more than ten years in the past (**Table 1**). On day 0, group 1 (2^nd^ HBvac NR) received a booster vaccination with the third-generation recombinant hepatitis B vaccine Sci-B-Vac™ (VBI Vaccines Inc., Rehovot, Israel), whereas group 2 (2^nd^ HBvac R) was revaccinated with the second-generation recombinant hepatitis B vaccine Twinrix^®^ (Glaxo Smith Kline, Brentford, UK). Peripheral blood was taken by venipuncture at baseline (day 0), and on day 7, and 28 after vaccination with the approval of the local ethic committees (School of Medicine, Technical University of Munich and Innsbruck Medical University). Data on age, weight, and height (BMI (body mass index) measurement), smoking status, alcohol consumption, medical co-morbidities, and medication were collected at baseline (**Table 1**). Informed consent was obtained from all participating individuals prior to their inclusion.

**Table 1:** Characteristics of study cohorts.

Cohorts (Number of participants)	Hepatitis B vaccine non-responders (n = 11)	Hepatitis B vaccine responders (n = 8)
Gender		
Female	n = 9	n = 6
Male	n = 2	n = 2
Mean age at 1. Visit (years)	25.7 (20.0 – 38.0)	30.0 (23.0 – 38.0)
Mean BMI (range)	24.7 (18.7 – 37.9)	24.6 (19.8 – 32.7)
Smoking (%)	18.2	not known
History of co-morbidities (%)	27.3	not known
Allergies (%)	54.5	not known

### 2.2 Determination of Serum Anti-HBs Levels

Serum levels of HBsAg-specific antibodies (anti-HBs) were quantified using the Architect^®^ chemiluminescence microparticle immunoassay (Abbott Diagnostics, Wiesbaden, Germany). The detection limit was 10 IU/mL.

### 2.3 Isolation and Cryoconservation of PBMC

Within 4 h after collection of heparinized whole blood human peripheral blood mononuclear cells (PBMC) were separated by Ficoll density gradient (human Pancoll, PAN-BIOTECH, Aidenbach, Germany) as described previously (40). PBMC were frozen in aliquots of 5 x 10^6^ PBMC per vial in 1.8 mL cryotubes (Thermo Scientific, Roskilde, Denmark) in a concentration of 1 x 10^7^ PBMC per 1 mL freezing medium (fetal calf serum (FCS) (Life Technologies, Darmstadt, Germany) supplemented with 10% DMSO (Sigma-Aldrich, Steinheim, Germany) in a freezing container (Mr. Frosty, Thermo Scientific, Roskilde, Denmark) and put on -80°C. After 48 h PBMC were stored in the vapor phase of a liquid nitrogen tank until further use.

### 2.4 Thawing and Resting of PBMC

PBMC were thawed at 37°C using CTL Anti-Aggregate Wash™ 20x Solution (Cellular Technology Limited (CTL) Europe, Bonn, Germany) diluted in RPMI-1640 medium (Life Technologies, Darmstadt, Germany) (1:20). Cells were counted with an automated cell counter (Vi-cell XR, Beckman Coulter, Krefeld, Germany) in CTL-Test™ Medium (Cellular Technology Limited (CTL) Europe, Bonn, Germany). The median cell recovery after thawing was 4.4 x 10^6^ PBMC per vial with a median viability of 92%. For a standard resting procedure PBMC were incubated for 18 h at 37°C in a humidified atmosphere at 5% CO_2_ in a concentration of 2 x 10^6^ PBMC/mL CTL-Test™ medium supplemented with 10% FCS and 1% penicillin–streptomycin (PenStrep, Life Technologies, Invitrogen, Darmstadt, Germany) (abbr.: RPMI-10) RPMI-10. The median cell recovery after resting was 4.2 x 10^6^ PBMC per vial with a median viability of 94%.

### 2.5 Flow Cytometry-Based Analysis of Cell Frequencies and Cytokine Production

#### 2.5.1 *Ex Vivo* Stimulation of PBMC

2 x 10^6^ overnight rested PBMC were seeded in polypropylene U-bottom 96-well microtiter plates (Fisher Scientific, Hampton, USA) with CTL-Test™ Medium and either stimulated with CpG oligodeoxynucleotides (10 µg/mL) (InvivoGen, Toulouse, France), PMA (Phorbol-12-myristat-13-acetat) (30 ng/mL) (Sigma-Aldrich, St. Louis, USA), and Ionomycin (1 µg/mL) (Sigma-Aldrich, St. Louis, USA) (referred to as “CpG+P/I”) or left unstimulated as a medium control to define background activity. After 1 h of incubation at 37°C in 5% CO_2_, 10 µg/mL of secretion blocker Brefeldin A (Sigma-Aldrich, St. Louis, USA) in a total volume of 50 µL CTL-Test™ medium was added to each well. Stimulation was stopped after 4 h by transferring the plates to 4°C overnight.

#### 2.5.2 Surface Marker and Intracellular Cytokine Staining

After a centrifugation step (560g, 5 min, 4°C), PBMC were resuspended in 100 µL of eBioscience™ Flow Cytometry Staining Buffer (Life Technologies, Invitrogen, Darmstadt, Germany) and a Fc block (Human TruStain FcX™, BioLegend, San Diego, USA, 1:20) was added followed by incubation for 10 min at RT. Next, PBMC were stained with 100 µL of UV Live/Dead working solution (1:100 LIVE/DEAD™ Fixable Blue Dead Cell Stain Kit (Life Technologies, Invitrogen, Darmstadt, Germany) for 30 min on ice in the dark followed by two washing steps with 200 µL eBioscience™ Flow Cytometry Staining Buffer (Life Technologies, Invitrogen, Darmstadt, Germany). For a combined intracellular and surface staining, PBMC were fixed for 20 min on ice in the dark using 100 µL/well eBioscience™ Intracellular Fixation buffer (Life Technologies, Invitrogen, Darmstadt, Germany) and afterwards centrifuged (710g, 5 min, 4°C) and washed in permeabilization buffer eBioscience (10X) (Life Technologies, Invitrogen, Darmstadt, Germany) for three times. PBMC were stained with the antibodies listed in Additional file 1: **Table S1** in a total volume of 100 µL antibody staining solution (1:2) (Permeabilization Buffer and Brilliant Stain Buffer (BD Biosciences, Franklin Lakes, USA) for 30 min on ice in the dark. Cells were washed twice with 200 µL/well Permeabilization Buffer and finally re-suspended in 300 µL Staining Buffer and Permeabilization Buffer (1:2) for acquisition. For compensation, UltraComp eBeads™ Compensation Beads (Life Technologies, Invitrogen, Darmstadt, Germany) and ArC™ Amine Reactive Compensation Bead Kit (Life Technologies, Invitrogen, Darmstadt, Germany) were stained with antibodies and live/dead discriminating dyes without fixation steps and finally re-suspended in 300 µL Staining and Permeabilization buffer (1:2) for acquisition. Cells were stored cold and in the dark until acquisition.

#### 2.5.3 Data Acquisition

Acquisition of samples was performed within 4 h after staining using a BD LSR Fortessa flow cytometer (Becton Dickinson, Franklin Lakes, USA) equipped with a 96-well plate reader and FACSDiva Software V.6.0 (Becton Dickinson, Heidelberg, Germany). On a weekly basis, the flow cytometer’s performance was checked and settings were configured with Cytometer, Setup & Tracking beads (Becton Dickinson, Franklin Lakes, USA). Photomultiplier voltages were adjusted with the help of unstained cells for all parameters.

#### 2.5.4 Gating Strategy

Flow cytometry-based analysis was performed on at least 1.0 x 10^5^ living lymphocytes using the software FlowJo version 10 (FlowJo LLC, BD, Ashland, USA). Gating strategy for analysis of *ex vivo* re-stimulated PBMC is shown in the supplementary information (Additional file 2: **Figure S1**). Each gate was set in the negative control sample and then adjusted to the antigen stimulated samples. In detail, we firstly set a broad forward-side scatter gate to prevent excluding cells of interest, followed by an exclusion of dead cells. Using a FSC-W against FSC-A plot, we excluded doublets. Next, we gated on CD3^-^CD14^-^ cells and CD19^+^ B cells. Within the CD19^+^ cells, we gated on CD24^+^CD27^+^ and CD24^high^CD38^high^ Breg, respectively. FMO controls were used to gate on CD24^+^, CD27^+^, and CD38^+^ cells, respectively. For functional analysis, we gated on IL-10, IL-35, and TGF-ß expression in the CD19^+^ B cell, CD24^+^CD27^+^, and CD24^high^CD38^high^ Breg population. Two independent audits were performed to control the gating.

#### 2.5.5 Data Interpretation

We detected significantly higher levels of IL-10, IL-35, and TGF-ß expression of CD24^+^CD27^+^ and/or CD24^high^CD38^high^ Breg upon CpG+P/I re-stimulation compared to unstimulated PBMC in both cohorts. In line with this, we used CpG+P/I induced frequencies for further analysis without further background subtraction. Detected frequencies of cytokine expressing Breg which derived from rare events (< 20 events) were excluded from further analysis to avoid misleading interpretations. The median fluorescence intensity (MFI) of CD24^+^CD27^+^IL-10^+^, CD24^+^CD27^+^IL-35^+^, CD24^+^CD27^+^TGF-ß^+^ and CD24^high^CD38^high^IL-10^+^ Breg of second-generation hepatitis B vaccine non-responders and responders before (day 0) and 7, and 28 days after vaccination with a third-generation hepatitis B vaccine are shown in Additional file 3: **Figure S2**.

### 2.6 Statistical Analysis

All results were included in the analysis, as no attempt was made to exclude outliers. All tests were two-sided and conducted on exploratory 5% significance levels. Effect measures are presented with 95% confidence intervals. Nonparametric statistical tests were applied in all cases. Unpaired Mann-Whitney U test was used to define significance of values between the 2^nd^ HBvac NR and 2^nd^ HBvac R group. One-Way ANOVA (Friedman test) was applied for analyses within the two groups for the different time points. In case of resulting *p*-values < 0.05, paired Wilcoxon signed rank tests were performed to assess significance of change in values within the 2^nd^ HBvac NR and 2^nd^ HBvac R group, respectively. The software Graph Pad Prism 9.1.0 (GraphPad Software, La Jolla, California, USA) was used for statistical analyses. Interpretation of Spearman`s correlation coefficient was performed according to Cohen (41).

## 3 Results

### 3.1 Vaccination With Third-Generation Hepatitis B Vaccine Sci-B-Vac Overcomes Non-Responsiveness to Second-Generation Hepatitis B Vaccination

**Figure 1:**
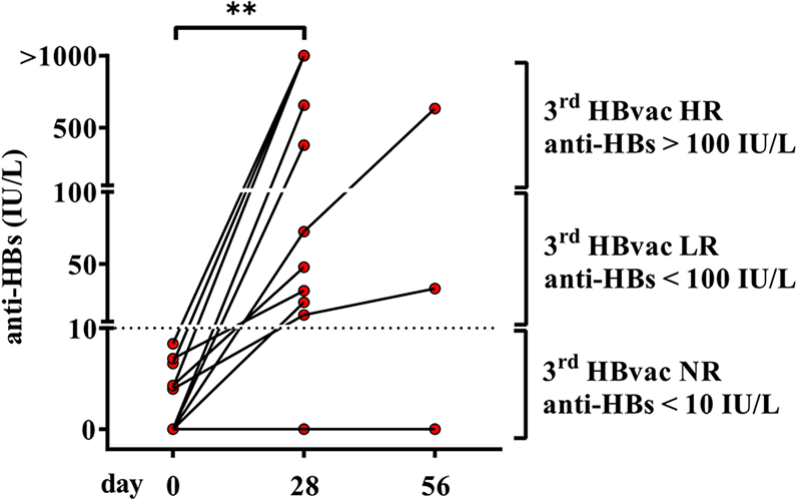
Anti-HBs levels of second-generation hepatitis B vaccine non-responders before and after vaccination with third-generation hepatitis B vaccine Sci-B-Vac^™^. Depicted are anti-HBs levels (IU/L) of second-generation hepatitis B vaccine non-responders (2^nd^ HBvac NR, n = 11) before (day 0), after primary (day 28), and second (day 56, n = 3) Sci-B-Vac™ vaccination. The dashed line indicates the threshold for protective anti-HBs levels (> 10 IU/L). The interconnected lines link the respective data points of each subject at each time point. Statistics are based on paired Wilcoxon signed rank tests. **p < 0.01.

First, we examined anti-HBs (antibody to hepatitis B surface antigen) levels of second-generation hepatitis B vaccine non-responders (n = 11) (referred to as “2^nd^ HBvac NR”) before (day 0) and after vaccination (day 28 and 56) with the third-generation hepatitis B vaccine Sci-B-Vac. Sci-B-Vac vaccination led to anti-HBs seroconversion (anti-HBs levels >Partner Site Munich 10 IU/L) in 10/11 (90.9%) 2^nd^ HBvac NR. In detail, we detected median anti-HBs levels of 3.97 IU/L (0Partner Site Munich - 8.46 IU/L) on day 0 and 72.44 IU/L (0 - >1000 IU/L) on day 28 after Sci-B-Vac vaccination (*p* = 0.002) (**Figure 1**). Three of six Sci-B-Vac vaccination non-(anti-HBs levels < 10) or low (anti-HBs levels < 100) -responder subjects (42) received a second Sci-B-Vac vaccination on day 30, resulting in anti-HBs levels of 0, 33.0, and 634.2 IU/L at day 56, respectively (**Figure 1**).

Comparison of the characteristics of Sci-B-Vac vaccine low- (“3^rd^ HBvac LR”; anti-HBs < 100 IU/L; n = 5) and high- (“3^rd^ HBvac HR”; anti-HBs > 100 IU/L; n = 5) responders revealed no differences in age (mean: 25.6 (range 21-31) and 23.4 (range 21-30) years, respectively) (*p* = 0.524), gender (4 female, 1 male in each cohort), smoking habits (one smoker in the 3^rd^ HBvac HR group), and in history of comorbidities (one person with comorbidities per group) and medication (none in each group).

The only one remaining non-responder from study group 1 (2^nd^ and 3^rd^ HBvac NR) had risk factors (smoking, obesity, hypertension) known to impair vaccine efficacy.

### 3.2 Vaccine Non-Responders Showed Higher Numbers of CD24^high^CD38^high^ Breg And Lower Breg-Derived IL-10 Expression Compared to Vaccine Responders

High levels of immunomodulatory lymphocytes and related cytokines such as regulatory B cells (Breg) and IL-10 have been associated with serological non-response to hepatitis B vaccination. To investigate this, we analyzed frequencies of two populations of Breg (CD24^+^CD27^+^ and CD24^high^CD38^high^ Breg) and respective IL-10 levels in a cohort of 2^nd^ HBvac NR (n = 11) at baseline visit and on day 7 and 28 post Sci-B-Vac booster vaccination. For comparative analysis, we additionally determined frequencies of Breg and IL-10 expression levels on day 0, 7, and 28 also in a cohort of second-generation hepatitis B vaccine responders (referred to as “2^nd^ HBvac R”; n = 8) receiving a second-generation hepatitis B booster vaccination.

**Figure 2:**
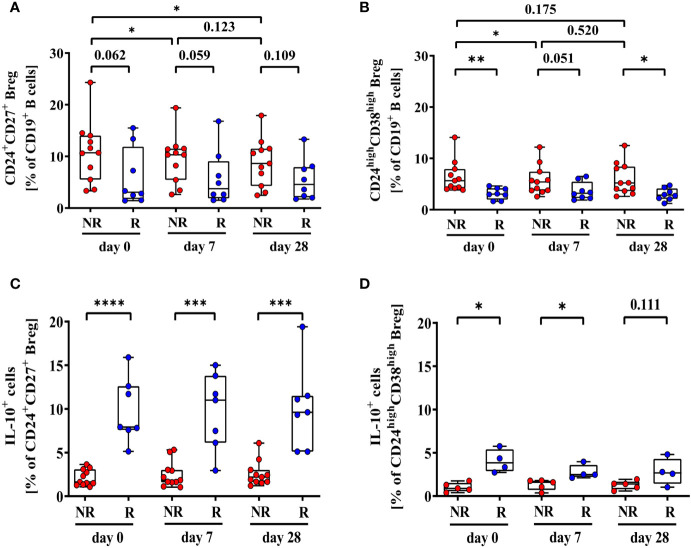
Frequencies of CD24^+^CD27^+^ **(A)**, CD24^high^CD38^high^ **(B)**, CD24^+^CD27^+^IL-10^+^ **(C)**, and CD24^high^CD38^high^IL-10^+^ Breg **(D)** of second-generation hepatitis B vaccine non-responders and responders. Depicted are (CpG+P/I)-induced frequencies of CD24^+^CD27^+^ **(A)**, CD24^high^CD38^high^ **(B)**, CD24^+^CD27^+^IL-10^+^ **(C)**, and CD24^high^CD38^high^IL-10^+^ Breg **(D)** of second-generation hepatitis B vaccine non-responders (NR, red circles, n = 11) and second-generation hepatitis B vaccine responders (R, blue circles, n = 8) before (day 0) and 7, and 28 days after vaccination with a third- (NR group) or second- (R group) generation hepatitis B vaccine, respectively. The boxes show the median and the 25^th^ and 75^th^ percentile. Whiskers indicate the highest and lowest values. Statistical analyses are based either on unpaired Mann-Whitney U tests for the analysis of the NR *vs.* the R group or on Friedman tests with post Wilcoxon signed rank testing for analysis of the impact of third- and second-generation hepatitis B vaccines within the NR or R group. *p < 0.05; **p < 0.01; ***p < 0.001; ****p < 0.0001.

Frequencies of CD24^+^CD27^+^ Breg tend to be higher in 2^nd^ HBvac NR compared to 2^nd^ HBvac R at all three time points (d0: *p* = 0.062; d7: *p* = 0.059; d28: *p* = 0.109) (**Figure 2A**). In parallel, we observed significantly higher frequencies of CD24^high^CD38^high^ Breg in the 2^nd^ HBvac NR compared to the 2^nd^ HBvac R group on day 0 (*p* = 0.004) and day 28 (*p* = 0.012), but not on day 7 (*p* = 0.051) (**Figure 2B** and **Table 2**).

**Table 2:** Frequencies and cytokine expression levels of CD24^+^CD27^+^ and CD24^high^CD38^high^ Breg and B10^+^ cells.

Cell population	Time point	2^nd^ HBvac NR; Median (min-max) (%)	2^nd^ HBvac R; Median (min-max) (%)	*p*-value
CD24^+^CD27^+^ Breg	d0	10.70 (3.33 – 24.3)	3.07 (1.44 – 15.50)	0.062
	d7	10.30 (2.62– 19.40)	3.75 (1.52 – 16.80)	0.059
	d28	8.62 (2.47 – 17.90)	4.56 (1.75 – 13.30)	0.109
CD24^high^CD38^high^ Breg	d0	5.67 (3.84 – 14.10)	3.04 (1.63 – 4.63)	0.004
	d7	5.42 (2.54 – 12.20)	3.22 (1.88 – 6.48)	0.051
	d28	5.20 (2.59 – 12.50)	2.84 (1.23 – 4.75)	0.012
CD24^+^CD27^+^IL-10^+^ Breg	d0	1.63 (1.08 – 3.63)	7.92 (5.14 – 15.90)	<0.0001
	d7	1.84 (1.04 – 5.31)	11.0 (2.95 – 15.0)	0.0004
	d28	2.19 (1.20 – 6.08)	9.61 (5.10 – 19.40)	0.0003
CD24^high^CD38^high^IL-10^+^ Breg	d0	0.89 (0.39 – 1.75)	3.85 (2.73 – 5.75)	0.016
	d7	1.59 (0.36 – 1.79)	2.48 (2.11 – 3.97)	0.016
	d28	1.39 (0.58 – 1.93)	2.68 (1.03 – 4.80)	0.111
CD24^+^CD27^+^IL-35^+^ Breg	d0	1.68 (0.48 – 2.14)	1.19 (0.76 – 2.54)	ND
	d7	1.39 (0.73 – 3.06)	1.05 (0.71 – 2.73)	ND
	d28	1.23 (0.52 – 3.94)	1.76 (0.70 – 2.34)	ND
CD24^+^CD27^+^TGF-ß^+^ Breg	d0	1.49 (0.78 – 3.20)	0.31	ND
	d7	1.34 (0.89 – 2.09)	0.66	ND
	d28	1.31 (0.79 – 3.21)	0.38	ND
CD24^high^CD38^high^TGF-ß^+^ Breg	d0	1.44 (0.77 – 1.85)	U	ND
	d7	1.07 (0.72 – 2.20)	U	ND
	d28	1.18 (0.60 - 2.44)	U	ND
B10^+^ cells	d0	0.70 (0.28 – 1.51)	2.24 (1.29 – 4.40)	<0.0001
	d7	0.66 (0.23 – 2.01)	2.13 (1.53 – 3.67)	0.0005
	d28	0.77 (0.24 – 1.75)	2.18 (1.21 – 3.92)	0.0001

U, undetectable; ND, not done.

Comparison of CpG- and PMA/Ionomycin (CpG+P/I)-induced IL-10 expression levels of Breg between both cohorts showed significantly lower frequencies of CD24^+^CD27^+^IL-10^+^ Breg in the 2^nd^ HBvac NR group at all time points (d0: *p* < 0.0001; d7: *p* = 0.0004; d28: *p* = 0.0003) (**Figure 2C**). Frequencies of CD24^high^CD38^high^IL-10^+^ Breg were also significantly lower in 2^nd^ HBvac NR compared to 2^nd^ HBvac R on day 0 and 7, but not on day 28 (d0: *p* = 0.016; d7: *p* = 0.016; d28: *p* = 0.111) (**Figure 2D** and **Table 2**).

**Figure 3:**
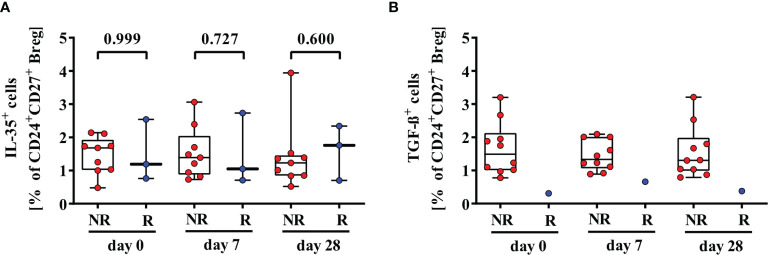
Comparison of frequencies of CD24^+^CD27^+^IL-35^+^ **(A)** and CD24^+^CD27^+^TGF-ß^+^ **(B)** Breg of second-generation hepatitis B vaccine non-responders and responders. Depicted are (CpG+P/I)-induced frequencies of CD24^+^CD27^+^IL-35^+^ **(A)** and CD24^+^CD27^+^TGF-ß^+^ **(B)** Breg of second-generation hepatitis B vaccine non-responders (NR, red circles) and second-generation hepatitis B vaccine responders (R, blue circles) before (day 0) and 7, and 28 days after vaccination with a third- (NR group) or second- (R group) generation hepatitis B vaccine, respectively. The boxes show the median and the 25^th^ and 75^th^ percentile. Whiskers indicate the highest and lowest values. Statistical analyses were done with unpaired Mann-Whitney U tests.

Besides IL-10, other cytokines like IL-35 and TGF-ß could mediate immunosuppressive function of Breg. CD24^+^CD27^+^IL-35^+^ Breg were detectable in nine 2^nd^ HBvac NR and three 2^nd^ HBvac R (**Figure 3A**). Median frequencies of CD24^+^CD27^+^IL-35^+^ Breg were comparable between both groups and time points, but reactivity rates in the 2^nd^ HBvac R group were too low for statistical analysis (**Figure 3A** and **Table 1**). CD24^high^CD38^high^IL-35^+^ Breg were largely undetectable in both groups (1/11 2^nd^ HBvac NR and 0/8 2^nd^ HBvac R; data not shown). Regarding TGF-ß expression, we detected CD24^+^CD27^+^TGF-ß^+^ Breg in ten 2^nd^ HBvac NR but only one 2^nd^ HBvac R (**Figure 3B** and **Table 2**). CD24^high^CD38^high^TGF-ß^+^ Breg were detectable in six 2^nd^ HBvac NR subjects with comparable frequencies at all time points, but in none of the 2^nd^ HBvac R subjects (**Table 2**).

Correlation analysis revealed no correlation of pre-vaccination levels of CD24^+^CD27^+^IL-10^+^ Breg and anti-HBs levels on day 28 (*r_s_* = 0.083), but a moderate correlation of pre-vaccination levels of CD24^high^CD38^high^IL-10^+^ Breg and anti-HBs levels on day 28 (*r_s_* = 0.300) (Additional file 4: **Figures S3A, B**).

### 3.3 Significant Lower Frequencies of B10^+^ Cells in Vaccine Non-Responders Compared to Responders

**Figure 4:**
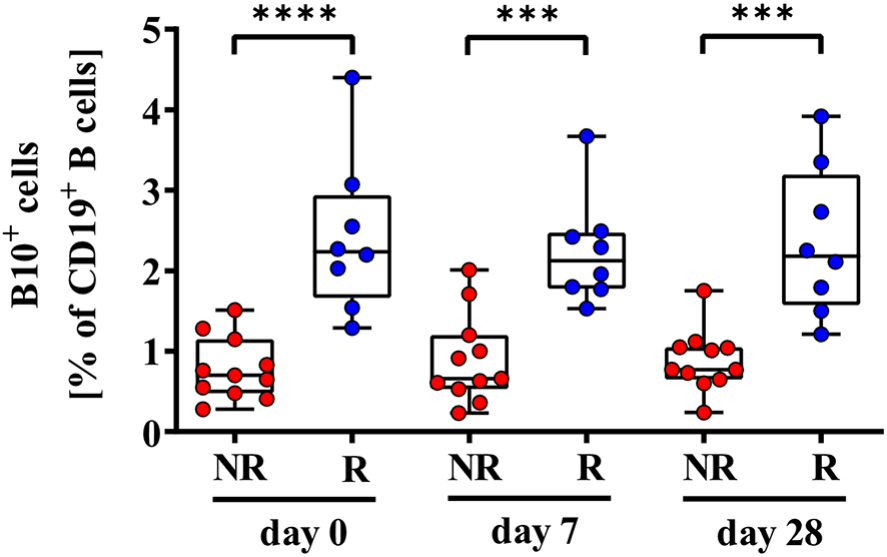
Comparison of frequencies of CD19^+^IL-10^+^ B (B10^+^) cells of second-generation hepatitis B vaccine non-responders and responders. Depicted are frequencies of B10^+^ cells of second-generation hepatitis B vaccine non-responders (NR, red circles) and second-generation hepatitis B vaccine responders (R, blue circles) before (day 0) and 7, and 28 days after vaccination with a third- (NR group) or second- (R group) generation hepatitis B vaccine, respectively. The boxes show the median and the 25^th^ and 75^th^ percentile. Whiskers indicate the highest and lowest values. Statistical analyses are based either on unpaired Mann-Whitney U tests for the analysis of the NR *vs*. the R group or on Friedman tests with post Wilcoxon signed rank testing for analysis of the impact of third- and second-generation hepatitis B vaccines within the NR or R group. ***p < 0.001; ****p < 0.0001.

We further analyzed CpG+P/I-induced IL-10 expression levels of CD19^+^ B cells (B10^+^ cells), since IL-10 plays an active role in B-cell differentiation into antibody secreting plasmablasts and B10^+^ cells. We determined significantly lower frequencies of B10^+^ cells in 2^nd^ HBvac NR compared to 2^nd^ HBvac R at baseline (day 0) (*p* < 0.0001), and also on day 7 (*p* = 0.0005), and day 28 (*p* = 0.0001) post vaccinations (**Figure 4**). In detail, we detected median frequencies of B10^+^ cells of 0.70% (0.28 – 1.51%) and 2.24% (1.29 – 4.40%) on day 0, 0.66% (0.23 – 2.01%), and 2.13% (1.53 – 3.67%) on day 7, and 0.77% (0.24 – 1.75%) and 2.18% (1.21 – 3.92%) on day 28 in 2^nd^ HBvac NR compared to 2^nd^ HBvac R, respectively (**Figure 4** and **Table 2**).

### 3.4 Frequencies of CD24^+^CD27^+^ and CD24^high^CD38^high^ Breg Decreased Upon Booster Vaccination With Third-Generation Vaccine but Remained Stable Upon Second-Generation Vaccine Booster Immunization

Next, we assessed the impact of booster vaccination with second- (2^nd^ HBvac R group) and third- (2^nd^ HBvac NR group) generation hepatitis B vaccines on Breg and B10^+^ cell frequencies as well as Breg-derived IL-10 expression

Upon booster vaccination with the third-generation vaccine (2^nd^ HBvac NR group), we observed significantly higher frequencies of CD24^+^CD27^+^ Breg prior (d0) compared to day 7 (*p* = 0.014) and day 28 (*p* = 0.032) post vaccination, but no difference between day 7 and 28 (*p* = 0.123) (**Figure 2A**). Frequencies of CD24^high^CD38^high^ Breg were also significantly higher prior vaccination (d0) compared to day 7 post vaccination (*p* = 0.045), but comparable between the other time points (d0 *vs*. d28: *p* = 0.175; d7 *vs*. d28: *p* = 0.520) (**Figure 2B**).

After booster vaccination with a second-generation vaccine (2^nd^ HBvac R group), frequencies of CD24^+^CD27^+^ and CD24^high^CD38^high^ Breg were comparable across time points (*p* = 0.531 and *p* = 0.236, respectively) (**Figures 2A, B**).

Analysis of CD24^+^CD27^+^IL-10^+^ and CD24^high^CD38^high^IL-10^+^ Breg numbers revealed no significant differences between the different booster vaccines and time points (2^nd^ HBvac NR: *p* = 0.643 and *p* = 0.124, respectively; 2^nd^ HBvac R: *p* = 0.423 and *p* = 0.125, respectively) (**Figures 2C, D**).

We also observed no significant differences in frequencies of B10^+^ cells between the different booster vaccines and time points (2^nd^ HBvac NR: *p* = 0.256 and 2^nd^ HBvac R: *p* = 0.967) (**Figure 4**).

### 3.5 Third-Generation Hepatitis B Vaccine Low- and High-Responders Showed Different Frequencies of CD24^+^CD27^+^ And CD24^high^CD38^high^ Breg Before and One Week After Booster Vaccination

**Figure 5:**
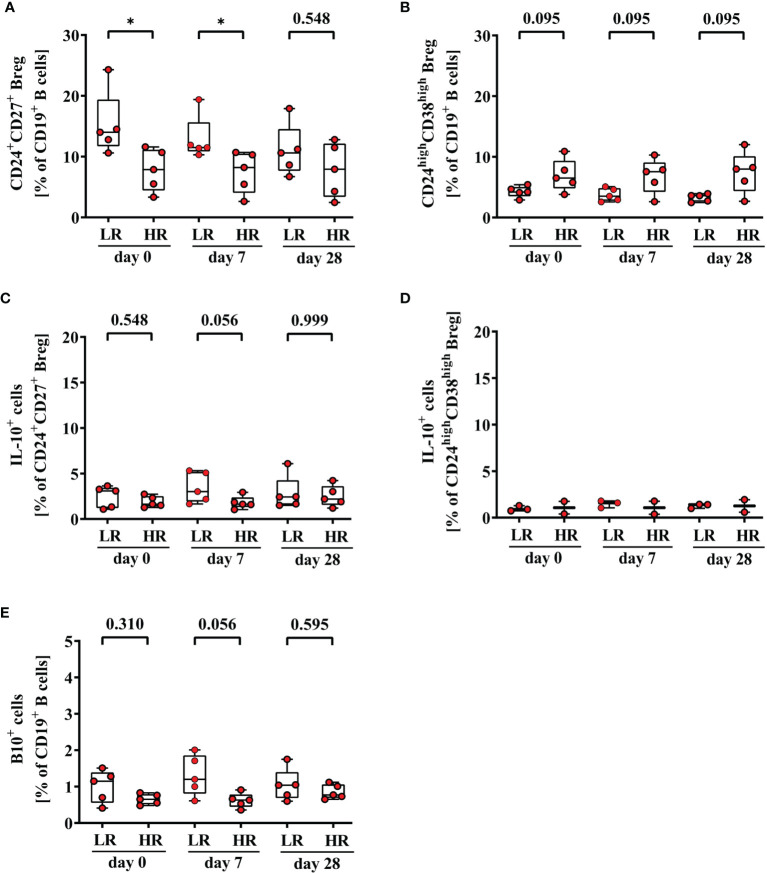
Frequencies of CD24^+^CD27^+^ **(A)**, CD24^high^CD38^high^ **(B)**, CD24^+^CD27^+^IL-10^+^ **(C)**, CD24^high^CD38^high^IL-10^+^ Breg **(D)**, and CD19^+^IL-10^+^ B (B10^+^) cells **(E)** of third-generation hepatitis B vaccine low- and high-responders. Depicted are (CpG+P/I)-induced frequencies of CD24^+^CD27^+^ **(A)**, CD24^high^CD38^high^ **(B)**, CD24^+^CD27^+^IL-10^+^ **(C)**, CD24^high^CD38^high^IL-10^+^ Breg **(D)** and CD19^+^IL-10^+^ B (B10^+^) cells **(E)** of third-generation hepatitis B vaccine low- (LR) and high-responders (HR) (red circles, n = 5, respectively) before (day 0) and 7, and 28 days after vaccination with a third-generation hepatitis B vaccine. The boxes show the median and the 25^th^ and 75^th^ percentile. Whiskers indicate the highest and lowest values. Statistical analyses are based on unpaired Mann-Whitney U tests. *p < 0.05.

Finally, we analyzed differences in frequencies of Breg and B10^+^ cells between third-generation hepatitis B vaccine low- (3^rd^ HBvac LR) and high-responders (3^rd^ HBvac HR) (n = 5, respectively). Frequencies of CD24^+^CD27^+^ Breg were significantly higher in the 3^rd^ HBvac LR compared to the 3^rd^ HBvac HR group on day 0 and day 7, but not on day 28 (d0: *p* = 0.032; d7: *p* = 0.024, and d28: *p* = 0.548, respectively) (**Figure 5A** and **Table 3**). In contrast, we observed a tendency of lower frequencies of CD24^high^CD38^high^ Breg in the 3^rd^ HBvac LR compared to the HR group at all three time points (*p* = 0.095, respectively) (**Figure 5B** and **Table 3**).

**Table 3:** Frequencies and cytokine expression levels of CD24^+^CD27^+^ and CD24^high^CD38^high^ Breg and B10^+^ cells of 3^rd^ HBvac LR and 3^rd^ HBvac HR.

Cell population	Time point	3^rd^ HBvac LR; Median (min-max) (%)	3^rd^ HBvac HR; Median (min-max) (%)	*p*-value
CD24^+^CD27^+^ Breg	d0	14.0 (10.60 – 24.30)	7.86 (3.33 – 11.60)	0.032
	d7	11.50 (10.30 – 19.40)	8.22 (2.62 – 10.70)	0.024
	d28	10.60 (6.72 – 17.90)	7.92 (2.47 – 12.80)	0.548
CD24^high^CD38^high^ Breg	d0	4.20 (2.90 – 5.42)	6.50 (3.81 – 10.90)	0.095
	d7	3.51 (2.57 – 5.09)	7.56 (2.61 – 10.30)	0.095
	d28	3.60 (2.46 – 3.93)	7.98 (2.66 – 12.0)	0.095
CD24^+^CD27^+^IL-10^+^ Breg	d0	3.09 (1.08 – 3.63)	1.63 (1.27 – 2.73)	0.548
	d7	3.02 (1.66 – 5.31)	1.63 (1.04 – 2.93)	0.056
	d28	2.42 (1.51 – 6.08)	2.19 (1.20 – 4.22)	0.999
CD24^high^CD38^high^IL-10^+^ Breg	d0	0.89 (0.73 – 1.29)	1.07 (0.39 – 1.75)	ND
	d7	1.59 (1.05 – 1.79)	1.07 (0.36 – 1.77)	ND
	d28	1.39 (0.99 – 1.42)	1.26 (0.58 – 1.93)	ND
B10^+^ cells	d0	1.15 (0.41 – 1.51)	0.65 (0.48 – 0.83)	0.310
	d7	1.20 (0.61 – 2.01)	0.63 (0.36 – 0.91)	0.056
	d28	1.04 (0.60 – 1.75)	0.77 (0.65 – 1.12)	0.595

ND, not done.

Regarding IL-10 expression levels, we observed a tendency of higher frequencies of B10^+^ cells (d0: *p* = 0.310; d7: *p* = 0.056; d28 *p* = 0.595) and CD24^+^CD27^+^IL-10^+^ Breg (d0: *p* = 0.548, d7: *p* = 0.056, d28: *p* = 0.999) in the 3^rd^ HBvac LR compared to the HR group at all three time points, respectively (**Figures 5C, E** and **Table 3**). Since CD24^high^CD38^high^IL-10^+^ Breg were only detectable in three 3^rd^ HBvac LR and two HR at all three time points, we could not conduct reliable statistical analyses between the two groups (**Figure 5D** and **Table 3**). One of the eleven 2^nd^ HBvac NR failed to produce protective anti-HBs levels even after vaccination with a third-generation hepatitis B vaccine. This 3^rd^ HBvac NR showed low frequencies of CD24^+^CD27^+^ and CD24^+^CD27^+^IL-10^+^ Breg (d0: 3.58%; d7: 3.44%; d28: 3.02% and d0: 1.49%; d7: 1.12%; d28: 1.65%, respectively). The frequencies of CD24^high^CD38^high^ Breg of the 3^rd^ HBvac NR subject were in the median range of all 2^nd^ HBvac NR subjects (d0: 5.67%; d7: 5.42%; d28: 5.20%). Noteworthy, the 3^rd^ HBvac NR subject showed the highest frequencies of CD19^+^ B cells within the 2^nd^ HBvac NR group, but the lowest frequencies of B10^+^ cells of all 2^nd^ HBvac NR and 2^nd^ HBvac R subjects (d0: 0.28%; d7: 0.23%; d28: 0.24%).

## 4 Discussion

Although several potential immunological mechanisms associated with hepatitis B vaccine non-responsiveness, like failure of antigen presentation or costimulatory signals, impact of certain HLA class II alleles, or lack of specific CD4 Th cells have been investigated, the decisive underlying immunological mechanisms remain unclear (10, 19, 20, 39, 43). In the current study, we asked whether non-responsiveness to hepatitis B vaccine is associated with a dysregulation of certain Breg subpopulations and analyzed their frequency and function in relation to second- *versus* third-generation hepatitis B vaccine induced immunity

Breg known to mediate immunosuppressive functions and the effect of different hepatitis B vaccine formulations on their frequency and function have not yet been investigated in parallel as possible factors involved in vaccine non-responsiveness. We performed comparative phenotypic and frequency analysis of CD24^+^CD27^+^ and CD24^high^CD38^high^ Breg in 2^nd^ in HBvac NR and R before and after booster vaccination to investigate whether non-responsiveness to hepatitis B vaccination is associated with alterations of Breg frequencies and their cytokine expression levels.

One of our main findings was the detection of higher frequencies of CD24^+^CD27^+^ and CD24^high^CD38^high^ Breg accompanied by lower levels of IL-10 expression in 2^nd^ HBvac NR compared to R. So far, very few studies investigated the association of Breg frequencies and IL-10 expression levels with non-responsiveness to hepatitis B vaccination (12, 38, 39). In contrast to our results, Bolther et al. observed no significant differences in frequencies of CD24^high^CD38^high^ Breg between 2^nd^ HBvac non-/low-responder and high-responder (38). But since the authors combined non- and low-responder in one group, the finding of comparable frequencies of CD24^high^CD38^high^ Breg in non-/low *versus* high-responder group could be biased by the very low number of non-responder subject included (38). Correlation analysis revealed no significant correlation of anti-HBs levels and frequencies of IL-10^+^CD24^high^CD38^high^ Breg, which is in line with our results (38).

In a study cohort of hepatitis B and tick-borne encephalitis (TBE) vaccine non-responders Garner-Spitzer et al. investigated, whether non-responsiveness is an antigen and/or vaccine-specific phenomenon. In line with our observations, Garner-Spitzer et al. reported clearly elevated frequencies of CD24^high^CD38^high^ Breg in HBvac NR pre- and post-booster TBE vaccination compared to TBE non- or high-responders (39). Since HBvac NR developed sufficient anti-TBE titers (neutralization test (NT) titers ≥ 1:10) and Breg frequencies did not decline post TBE booster vaccination, they concluded that the underlying immunological mechanisms of non-responsiveness rather depend on the applied vaccine antigen and host-related genetic predispositions (39).

Interestingly, TBE non- and high responders had comparable median frequencies of CD24^high^CD38^high^ Breg pre-booster which only increased in the non-responder group after TBE booster vaccination. In our study, we observed a decline of both, CD24^+^CD27^+^ and CD24^high^CD38^high^ Breg frequencies, post third-generation booster vaccination in 2^nd^ HBvac NR, which could indicate a positive effect of third-generation hepatitis B vaccines on Breg-mediated immunomodulation in HBvac NR. Interestingly we did not observe decreased numbers of CD24^+^CD27^+^ and CD24^high^CD38^high^ Breg post-boost with the second-generation hepatitis B vaccine.

Breg modulate immune responses predominantly, although not exclusively, *via* IL-10, therefore IL-10 expression is often used as a marker for Breg function (25). IL-10, a pleiotropic cytokine, is known to act as an immunoregulatory molecule which inhibits the production of inflammatory cytokines by T cells and monocytes and suppresses antigen-presentation e.g. by downregulation of MHC II expression (12, 44), but it is also known to promote B-cell differentiation into antibody secreting plasmablasts (12, 44–46). Several subsets of B cells produce IL-10, including B10^+^ cells, CD24^+^CD27^+^ and CD24^high^CD38^high^ Breg (30, 34). Within our study cohorts, we observed significantly lower IL-10 expression levels of CD24^+^CD27^+^ and CD24^high^CD38^high^ Breg and CD19^+^ B10^+^ cells in HBvac NR compared to HBvac R pre-booster.

Heine et al. reported that genes associated with the differentiation into antibody-secreting cells are upregulated in IL-10-secreting B cells and concluded that autocrine and paracrine IL-10 signaling contributes to differentiation of B cells into antibody secreting cells (45). Therefore, low IL-10 expression levels may additionally favor non-responsiveness to hepatitis B vaccination.

The fact, that we observed significantly lower IL-10 expression levels of CD24^+^CD27^+^ and CD24^high^CD38^high^ Breg and B10^+^ cells in 2^nd^ HBvac NR support the hypothesis that IL-10 may contribute to B-cell differentiation into anti-HBs secreting plasma cells (12). Of note, also the HBvac NR who was the only individual without protective anti-HBs titer even after booster with third-generation hepatitis B vaccine had very low frequencies of IL-10 expressing CD24^+^CD27^+^ and CD24^high^CD38^high^ Breg and B10^+^ cells. However, we could not observe a significant increase in IL-10 expression levels of CD24^+^CD27^+^ and CD24^high^CD38^high^ Breg in 2^nd^ HBvac NR who seroconverted after third-generation booster vaccination.

Although the immunoregulatory role of Breg is predominantly driven by IL-10, other cytokines, like IL-35 and TGF-ß are known to impact responsiveness to hepatitis B vaccination (10, 26–29). Breg-derived TGF-β can lead to an expansion of Treg linked with impaired antibody production (26, 35). In line with reports of Jarrosson et al. (10), we observed robust frequencies of IL-35 and TGF-ß expressing CD24^+^CD27^+^ Breg in the 2^nd^ HBvac NR whereas in 2^nd^ HBvac R group, these Breg were mostly undetectable.

Another important result of our study was the high seroconversion rate of HBvac NR after booster vaccination with a third-generation hepatitis B vaccine which was also observed in previous studies, using Sci-B-Vac^™^ (9, 47–49). Commonly used hepatitis B vaccines belong to the second-generation vaccines and are composed of the yeast-derived recombinant non-glycosylated small (S) HBV envelope protein (HBsAg) (3), whereas third-generation hepatitis B vaccines like Sci-B-Vac^™^, consist of glycosylated and non-glycosylated pre-S1, pre-S2, and S proteins produced in mammalian cells (48). Differences in glycosylation patterns and physical properties of these vaccine antigens might result in higher immunogenicity and successful induction of protective anti-HBs titers (9, 47, 49–51). The exact underlying immunological mechanisms of higher anti-HBs seroconversion rates in formerly non-responders are still unclear, but it is assumed that the additional pre-S1 and pre-S2 domains increase immunogenicity and may evade genetic non-responsiveness to the S antigen (52, 53).

As postulated by Garner-Spitzer et al., non-responder status does probably not reflect a basic defect in antibody production, as HBV-NR respond well to TBE and influenza vaccine. Our data support this hypothesis since ten of eleven 2^nd^ HBvac NR showed a seroconversion after vaccination with Sci-B-Vac^™^. There are reports suggesting that a real hepatitis B non-responder status is quite rare and that most non-responders are indeed low-responders where higher vaccine doses, intradermal vaccine application or repeated vaccinations could overcome the NR status (54, 55). At least for our study cohort this does not apply, as most study participants already received several booster vaccinations with second-generation vaccines without showing seroconversion. The fact, that vaccination with a third-generation hepatitis B vaccine lead to anti-HBs seroconversion in more than 90% of our study participants also argues against an overall defect in the priming and generation of vaccine-induced memory B cells. Valats et al. reported that 2^nd^ HBvac NR showed substantial numbers of HBsAg-specific memory B cells able to differentiate into anti-HBs secreting plasma cells upon *in vitro* re-stimulation (54). Since production of anti-HBs is known to be Th cell-dependent (19, 56–59) also a dysfunction in T-cell help mediating hepatitis B vaccine non-responsiveness was discussed (60, 61). But this is contradicted by the fact that several studies reported vaccine-induced, HBs-Ag-specific T cells being detectable in second-generation hepatitis B vaccine non-responders (10, 62). We have not investigated Th cell function or immunoregulatory T-cell subsets in our study cohorts as our main focus was the possible involvement of Breg on vaccine non-responsiveness. It remains to be investigated whether and how Breg-mediated effects described here interact with other immunoregulatory mechanisms (e.g. a vaccine-induced, dysfunctional T-cell response) which might synergistically favor hepatitis B vaccine non-responsiveness.

In summary, we report significantly higher frequencies of CD24^high^CD38^high^ Breg in parallel with significantly lower IL-10 expression levels of CD24^+^CD27^+^ and CD24^high^CD38^high^ Breg in 2^nd^ HBvac NR compared to 2^nd^ HBvac R. Anti-HBs seroconversion accompanied by a decrease of Breg numbers after booster immunization with a third-generation hepatitis B vaccine could indicate a positive effect of third-generation hepatitis B vaccines on Breg-mediated immunomodulation in hepatitis B vaccine non-responders. Further studies investigating Breg-mediated mechanisms involved in non-responsiveness to vaccines also during primary series of hepatitis B vaccination may help to further improve immunogenicity of existing or the development of new vaccines to overcome non-responsiveness to hepatitis B vaccines.

## Data Availability Statement

The raw data supporting the conclusions of this article will be made available by the authors, without undue reservation.

## Ethics Statement

The studies involving human participants were reviewed and approved by ethic committees: School of Medicine, Technical University of Munich and Innsbruck Medical University. The patients/participants provided their written informed consent to participate in this study.

## Author Contributions

NK, TB, and UP conceived and designed the experiments. BW and BG-L provided blood samples. NK and LP performed the experiments and acquired the data. NK, LP, and TB analyzed the data. NK, LP, BW, BG-L, PK, HR, UP, and TB interpreted the data. NK, LP, and TB drafted the manuscript. All authors contributed to the article and approved the submitted version.

## Funding

This study was supported by Helmholtz Initiative for Personalized Medicine (iMed, project title: “Molecular basis and early predictors of non-responsiveness to hepatitis B vaccination”).

The research leading to these results has received funding from the European Union’s Seventh Framework Programme [FP7/2007-2013] under Grant Agreement No: 280873 ADITEC.

## Conflict of Interest

UP received funding for the project from the Helmholtz Association in context of the iMed initiative and the Helmholtz-Alberta-Initiative in Infectious Disease Research. UP serves as scientific advisor for Gilead, Leukocare, Abbvie, MSD, Arbutus, GSK, Vaccitech, Biontech, and Dicerna.

The remaining authors declare that the research was conducted in the absence of any commercial or financial relationships that could be construed as a potential conflict of interest.

## Publisher’s Note

All claims expressed in this article are solely those of the authors and do not necessarily represent those of their affiliated organizations, or those of the publisher, the editors and the reviewers. Any product that may be evaluated in this article, or claim that may be made by its manufacturer, is not guaranteed or endorsed by the publisher.

## Abbreviations

aa, amino acid; ANOVA, analysis of variance; anti-HBs, antibody to hepatitis B surface antigen; APC, antigen presenting cells; BMI, body mass index; Breg, regulatory B cells; CD, cluster of differentiation; CpG, Cytosine and Guanine oligonucleotides; CpG+P/I, CpG+PMA and Ionomycin; DMSO, dimethyl sulfoxide; FCS, fetal calf serum; FSC, forward scatter; HBsAg, hepatitis B surface antigen; HBV, hepatitis B virus; ICS, intracellular cytokine staining; IFN, interferon; IL, interleukin; MFI, median fluorescence intensity; MHC, major histocompatibility complex; PBMC, peripheral blood mononuclear cells; PBS, phosphate buffered saline; PMA, Phorbol-12-myristat-13-acetat; RPMI, Roswell Park Memorial Institute; RT, room temperature; TBE, tick-borne encephalitis; TGF-ß, Transforming growth factor beta; Th cells, T helper cells; TNF, tumor necrosis factor Treg, regulatory T cells; WHO, World Health Organization; 2nd HBvac NR, second-generation hepatitis B vaccine non-responder; 2nd HBvac R, second-generation hepatitis B vaccine responder; 3rd HBvac HR, third-generation hepatitis B vaccine high-responder; 3^rd^ HBvac LR, third-generation hepatitis B vaccine low-responder.
