# Supplementary material for: Hepatitis B Vaccine Non-Responders Show Higher Frequencies of CD24highCD38high Regulatory B Cells and Lower Levels of IL-10 Expression Compared to Responders
Source: Front Immunol. 2021 Sep 10;12:713351. doi: 10.3389/fimmu.2021.713351 (PMC8461011; doi:10.3389/fimmu.2021.713351)
Supplement: Supplementary file 1 [file Image_1.pdf]

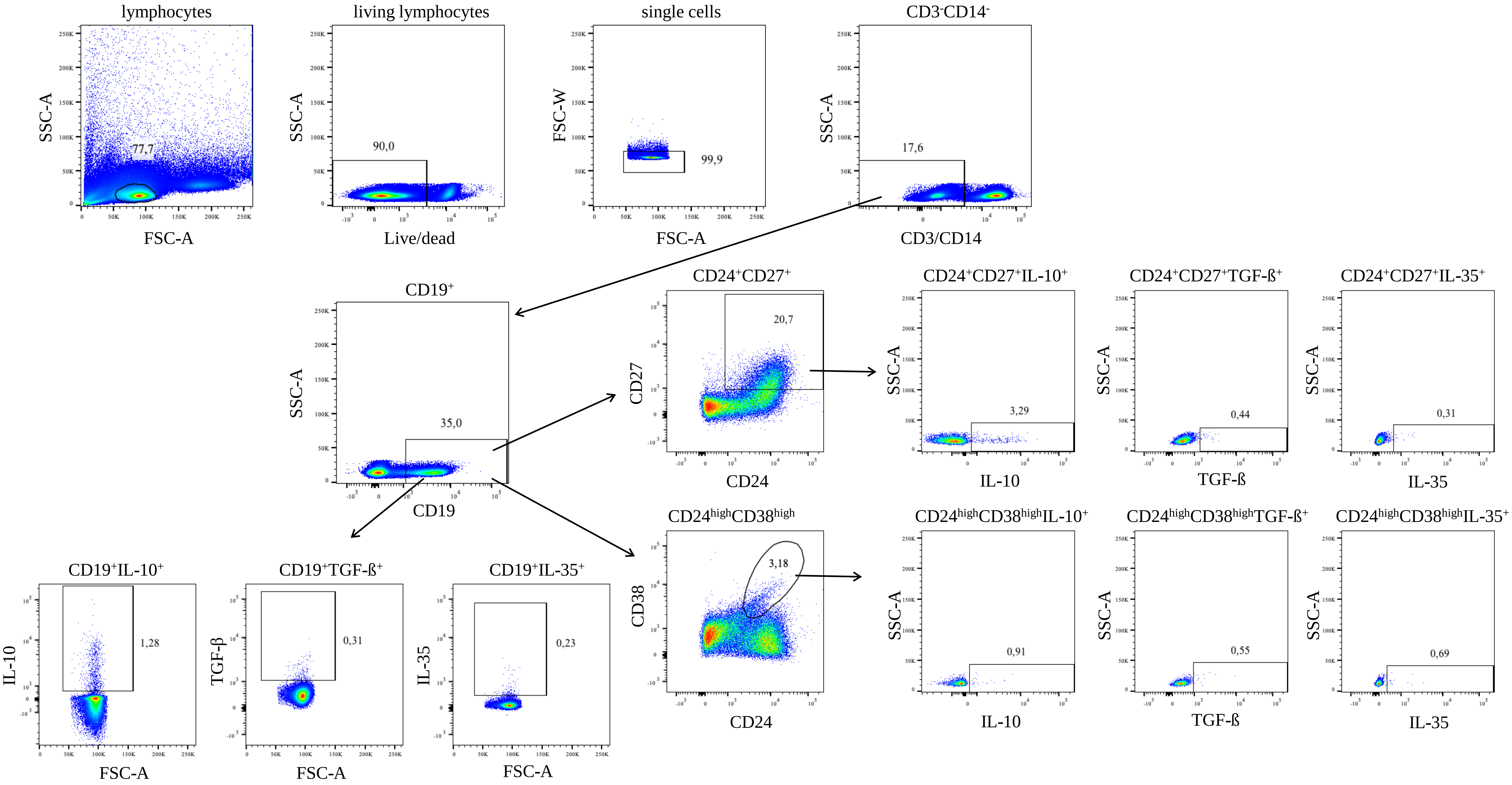

Additional file 2: Figure S1: Representative gating strategy for phenotypical analysis of Breg. Flow cytometry plots showing the gating strategy applied to determine CD19$^+$ cells, Breg subpopulations and the respective cytokine expression. Markers used to define each gate are indicated. Arrows between plots indicate subgating.
