# Supplementary figures and images for: Hepatitis B Vaccine Non-Responders Show Higher Frequencies of CD24highCD38high Regulatory B Cells and Lower Levels of IL-10 Expression Compared to Responders
Source: Front Immunol. 2021 Sep 10;12:713351. doi: 10.3389/fimmu.2021.713351 (PMC8461011; doi:10.3389/fimmu.2021.713351)

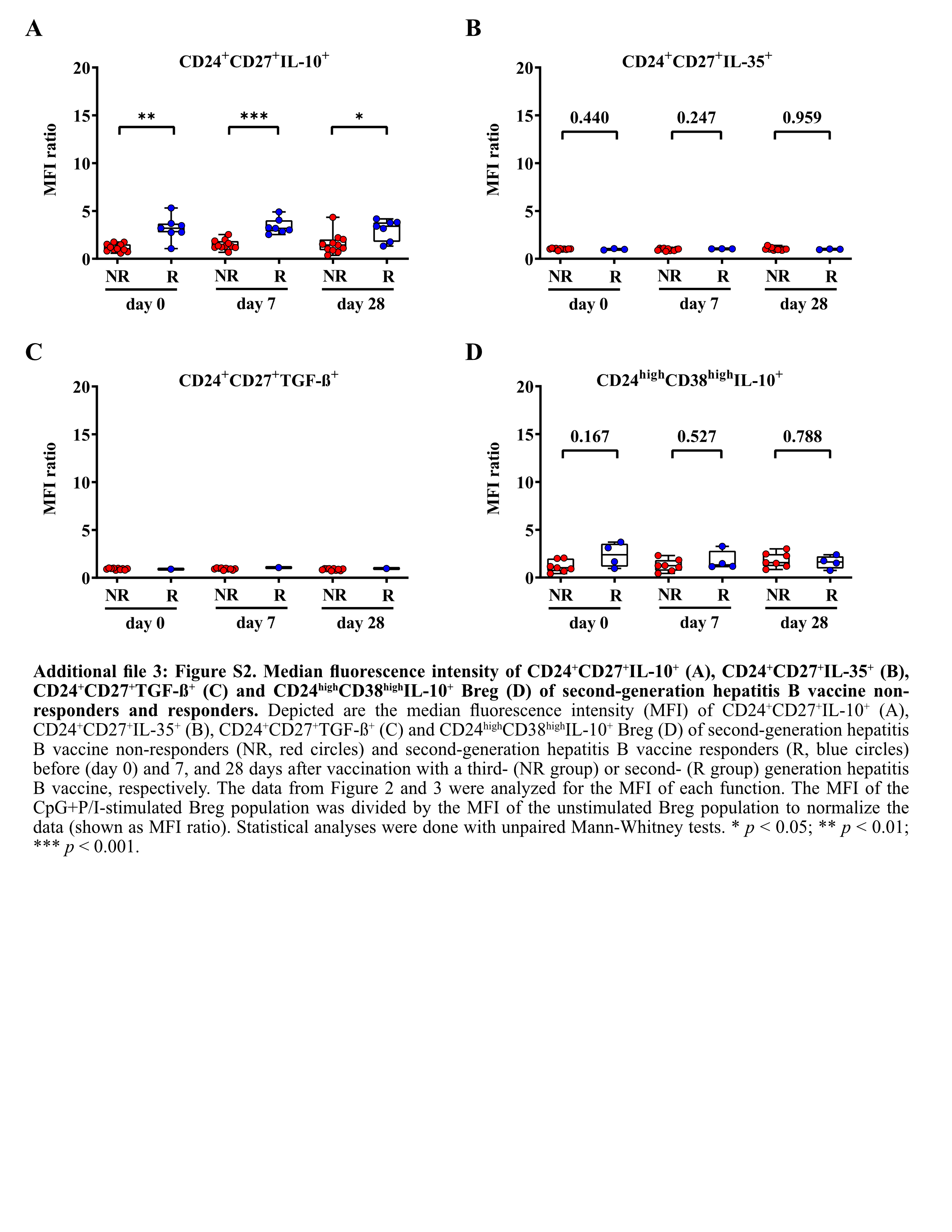

Supplement: Supplementary file 2 [file Image_2.jpeg]

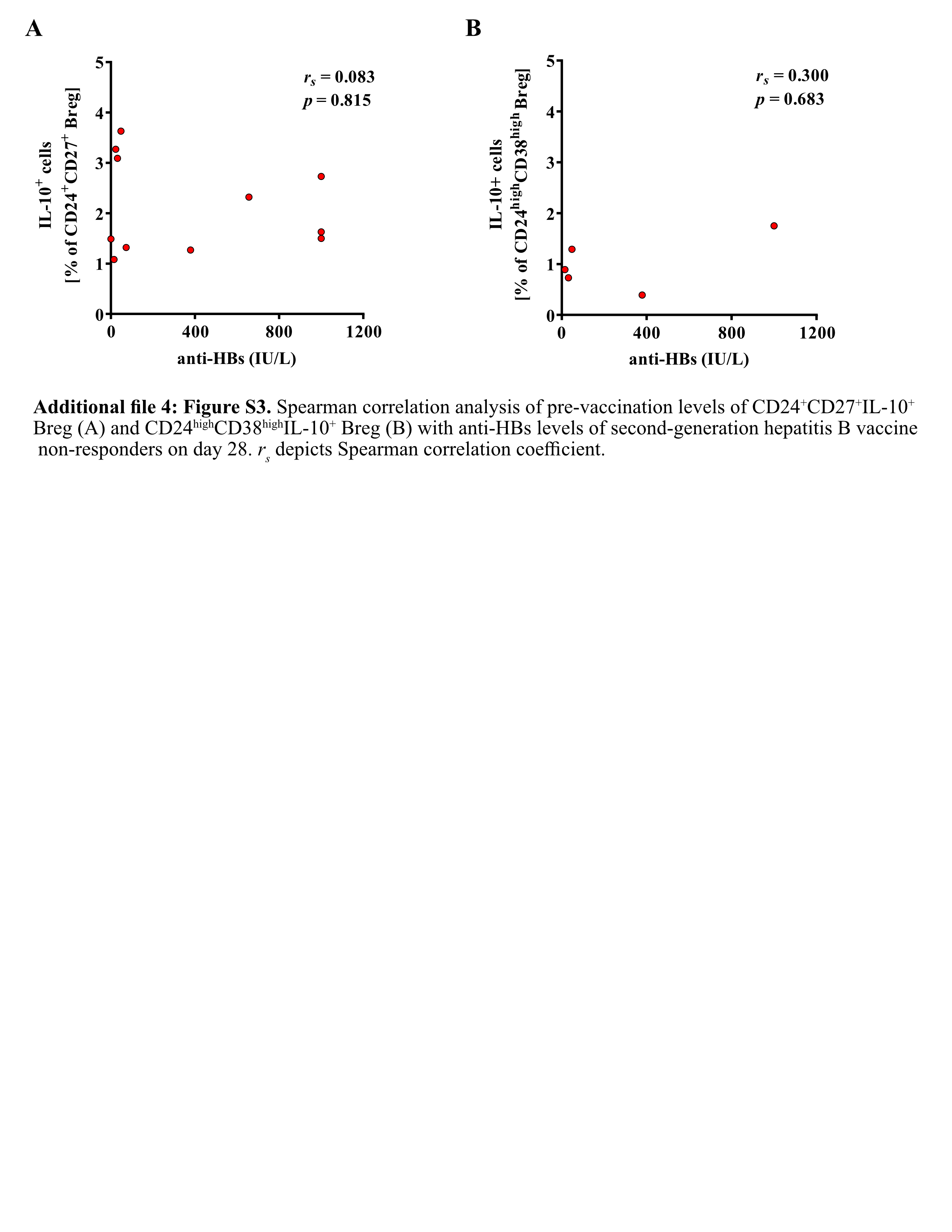

Supplement: Supplementary file 3 [file Image_3.jpeg]
