# Supplementary material for: Hepatitis B Vaccine Non-Responders Show Higher Frequencies of CD24highCD38high Regulatory B Cells and Lower Levels of IL-10 Expression Compared to Responders
Source: Front Immunol. 2021 Sep 10;12:713351. doi: 10.3389/fimmu.2021.713351 (PMC8461011; doi:10.3389/fimmu.2021.713351)
Supplement: Supplementary file 4 [file Table_1.pdf]

**Additional file 1: Table S1. Antibodies used for surface and intracellular cytokine staining**

| **Antibody** | **Clone** | **Assay concentration (μg/mL)** | **Company** |
|---|---|---|---|
| CD3-BUV395 | SK7 | 0.8 | BD Biosciences |
| CD14-BUV395 | MφP9 | 2.5 | BD Biosciences |
| CD19-BV421 | HIB19 | 1.3 | BioLegend |
| CD24-BV605 | ML5 | 3.0 | BioLegend |
| CD27-BV480 | L128 | 5.0 | BD Biosciences |
| CD38-BV785 | HIT2 | 5.3 | BioLegend |
| IL-10-PE-Dazzle594 | JES3-19F1 | 1.5 | BioLegend |
| EBI3 (IL-27 subunit)-APC (IL-35) | ebic6 | 0.3 | Invitrogen |
| LAP (TGF-β)-FITC | TW4-2F8 | 10.0 | BioLegend |
